# Evaluation of the prognostic value of computed tomography‐derived body composition in patients undergoing endovascular aneurysm repair

**DOI:** 10.1002/jcsm.13262

**Published:** 2023-05-23

**Authors:** Nicholas A. Bradley, Amy Walter, Ross Dolan, Alasdair Wilson, Tamim Siddiqui, Campbell S.D. Roxburgh, Donald C. McMillan, Graeme J.K. Guthrie

**Affiliations:** ^1^ University of Glasgow Glasgow UK; ^2^ NHS Tayside Dundee UK; ^3^ NHS Grampian Aberdeen UK; ^4^ NHS Lanarkshire Glasgow UK

**Keywords:** EVAR, AAA, Sarcopenia, SMI, Skeletal muscle index, Body composition analysis

## Abstract

**Background:**

Endovascular aneurysm repair (EVAR) is the most common mode of repair of abdominal aortic aneurysms (AAA) in the UK. EVAR ranges from standard infrarenal repair to complex fenestrated and branched EVAR (F/B‐EVAR). Sarcopenia is defined by lower muscle mass and function, which is associated with inferior perioperative outcomes. Computed tomography‐derived body composition analysis offers prognostic value in patients with cancer. Several authors have evaluated the role of body composition analysis in predicting outcomes in patients undergoing EVAR; however, the evidence base is limited by heterogeneous methodology.

**Methods:**

Six hundred seventy‐four consecutive patients (58 (8.6%) female, mean (SD) age 74.4 (6.8) years) undergoing EVAR and F/B‐EVAR at three large tertiary centres were retrospectively recruited. Subcutaneous and visceral fat indices (SFI and VFI), psoas and skeletal muscle indices, and skeletal muscle density were measured at the L3 vertebral level from pre‐operative computed tomographies. The maximally selected rank statistic technique was used to define optimal thresholds to predict mortality.

**Results:**

There were 191 deaths during the median follow‐up period of 60.0 months. Mean (95% CI) survival in the low SMI versus high SMI subgroups was 62.6 (58.5–66.7) versus 82.0 (78.7–85.3) months (*P* < 0.001). Mean (95% CI) survival in the low SFI versus high SFI subgroups was 56.4 (48.2–64.7) versus 77.1 (74.2–80.1) months (*P* < 0.001). One‐year mortality in the low SMI versus high SMI subgroups was 10% versus 3% (*P* < 0.001). Low SMI was associated with increased odds of one‐year mortality (OR 3.19, 95% CI 1.60–6.34, *P* < 0.001). Five‐year mortality in the low SMI versus high SMI subgroups was 55% versus 28% (*P* < 0.001). Low SMI was associated with increased odds of five‐year mortality (OR 1.54, 95% CI 1.11–2.14, *P* < 0.01). On multivariate analysis of all patients, low SFI (HR 1.90, 95% CI 1.30–2.76, *P* < 0.001) and low SMI (HR 1.88, 95% CI 1.34–2.63, *P* < 0.001) were associated with poorer survival. On multivariate analysis of asymptomatic AAA patients, low SFI (HR 1.54, 95% CI 1.01–2.35, *P* < 0.05) and low SMI (HR 1.71, 95% CI 1.20–2.42, *P* < 0.01) were associated with poorer survival.

**Conclusions:**

Low SMI and SFI are associated with poorer long‐term survival following EVAR and F/B‐EVAR. The relationship between body composition and prognosis requires further evaluation, and external validation of the thresholds proposed in patients with AAA is required.

## Introduction

Abdominal aortic aneurysm (AAA) is defined as pathological dilatation of the aorta to greater than 1.5 times the normal diameter. The estimated UK prevalence is 1.5%, rising to 1.9–4.0% in males aged over 65.^1^ Asymptomatic AAA may be identified incidentally during investigation for other conditions, or by the NHS AAA Screening Programme, which performs ultrasonographic assessment in males upon turning 65.^2^ Ruptured AAA is a terminal event if left untreated, with a 40–50% inpatient mortality. Larger diameter AAA carry increased risk of rupture, with data from the UK Small Aneurysm Trial supporting current practice of consideration of intervention in AAA over 5.5 cm.^3^

Options for the treatment of AAA include open surgical repair (OSR), or endovascular aneurysm repair (EVAR). OSR involves opening of the aneurysm sac and sutured interposition of a prosthetic graft. EVAR consists of delivery of a modular stent‐graft system within the aneurysm to exclude the aneurysm sac from the circulation, thereby reducing the risk of rupture. EVAR is performed for standard infrarenal AAA; and for more complex AAA which may involve or are adjacent to the ostia of the visceral vessels, these ‘complex aneurysms’ can be repaired using fenestrated and branched endografts (F/B‐EVAR).

The choice of OSR versus endovascular procedure is dependent on both patient and aneurysm characteristics. The recent 15‐year follow‐up of the EVAR‐1^4^ study showed an early (0–6 months) survival benefit in the EVAR cohort, but a late (beyond 8 years) increased risk in mortality in these patients, likely due to reintervention and secondary rupture.^4^ These findings were supported by a recent meta‐analysis of these 4 trials.^5^ In the UK, NICE recommends that for patients requiring planned intervention for AAA, those patients without abdominal co‐pathology, excessive anaesthetic risk, and/or medical comorbidities should be offered OSR as a first line intervention.^6^ EVAR is recommended for patients not meeting these criteria. In ruptured AAA with suitable aneurysm morphology, EVAR is recommended ahead of OSR, particularly in male patients over 70. The 2019 National Vascular Registry Annual Report confirmed EVAR as the predominant modality for repair of AAA with 63.4% of UK aneurysms managed by an endovascular approach.^7^

Sarcopenia is characterized by progressive loss of skeletal muscle volume and progressive reduction in skeletal muscle function (EWGSOP2 definition), and is associated with increasing age and chronic illness.^8^ Patients with sarcopenia are typically frail with associated poor functional status and inferior physiological reserve.^8^, ^9^ There is a well‐defined relationship between inferior perioperative outcomes and sarcopenia in a variety of surgical specialties.^9^, ^10^ Clinical assessment of sarcopenia can be readily performed, for example through assessment of grip strength or gait speed. In recent years, the use of body composition analysis has expanded as a technique for defining sarcopenic status. Typically, this involves the assessment of cross‐sectional imaging (computed tomography [CT] or magnetic resonance imaging) to measure muscle and fat areas,^11^, ^12^, ^13^, ^14^ which can be normalized to height^2^ to generate muscle and fat indices. Psoas and skeletal muscle indices (PMI and SMI) are associated with inferior survival outcomes, post‐operative complications, and increased length of stay, in a range of elective and emergency surgical procedures.^11^ CT‐derived skeletal muscle attenuation/density (SMD) has been used as a marker of intramuscular fat deposition (myosteatosis) and is thought to have a role alongside SMI to quantify muscle function in addition to volume, in line with the contemporary EWGSOP2 definition.^15^ The effect of visceral and subcutaneous fat indices (VFI and SFI) on prognosis has been investigated in patients with cancer.^16^

Body composition analysis is, to date, limited to a research tool though the clinical application is expanding. One key limitation is defining the threshold of values to define patients as sarcopenic. Several authors have defined thresholds in cohorts of patients with cancer,^17^, ^18^ though these thresholds have not yet been applied to patients with AAA.

A relationship between pre‐operative body composition analysis and clinical outcomes following EVAR has been reported, with some muscle parameters appearing to predict inferior post‐operative survival,^19^, ^20^ though the evidence base is more limited than in patients with cancer. Studies in patients with AAA are limited by short duration of follow‐up and consist largely of patients receiving EVAR for infrarenal asymptomatic AAA; there is a paucity of data assessing the importance of sarcopenia in patients undergoing complex F/B‐EVAR and in emergency cases. The literature to date is also limited by heterogeneous methods of assessment, lack of standardized thresholds, and choice of body composition parameter measured. This study aims to describe the prognostic value of body composition analysis of pre‐operative CT images in patients undergoing EVAR and F/B‐EVAR for AAA.

## Patients and methods

Patients were retrospectively identified from theatre records at three large tertiary referral centres in Scotland, UK, representing cases drawn from three health boards (NHS Grampian, NHS Lanarkshire, NHS Tayside). Specific procedural technique and choice of stent‐graft were at the discretion of each institution, though practice was broadly similar between sites throughout the study period. Consecutive cases undergoing EVAR or F/B‐EVAR to treat aortic aneurysmal disease between 1 January 2015 and 10 January 2021 were included. This period of study was chosen to reflect contemporary surgical practice and have a minimum follow‐up of 18 months. Patients with isolated iliac aneurysms, aortic dissection, penetrating aortic ulcer, incomplete clinical or follow‐up data, with corrupted CT images, and where height was unavailable for normalization, were excluded. Clinical, demographic, and outcome data were recorded from electronic case records; comorbidities were defined as per patients' community health records. Patients were sub‐grouped based on age (<65, 65–75, and >75), and procedure priority (asymptomatic AAA, symptomatic AAA, and ruptured AAA). Haemoglobin and creatinine were recorded as pre‐operative factors which may influence the outcome following surgery. Low haemoglobin was defined as <130 g/L (male patients) and <120 g/L (female patients), high creatinine was defined as >106 μmol/L (male patients) and >80 μmol/L (female patients), both based on local laboratory values. The primary outcome of interest was overall survival during the follow‐up period. West of Scotland Research Ethics Committee approval was obtained for this study (Reference 21/WS/0146; approval granted 23/11/2021).

Body composition analysis was performed using established methodology, previously described by Dolan et al.^21^ Pre‐operative CTs performed within 6 months before surgery (in keeping with current institutional practice) were accessed. The L3 vertebral level was identified using anatomical landmarks and the individual slice at the midpoint of the vertebral body was used for body composition analysis. Subcutaneous fat area, visceral fat area, psoas muscle area, total skeletal muscle area, and skeletal muscle density were manually measured using ImageJ v1.53 software. In keeping with widely accepted values,^22^ for muscle tissue thresholds of −29 to +150 Hounsfield Units were applied, and for adipose tissue thresholds of −190 to −30 Hounsfield Units were applied. The areas obtained were normalized to height^2^. SFI, VFI, PMI, SMI, and SMD were recorded. All measurements were performed systematically by one researcher (NAB). A random 15% sample of images were then analysed independently by a second researcher who has previous peer reviewed publication with this methodology (RD).^21^ An intraclass correlation coefficient between the two researchers' results was calculated as 0.992, indicating an excellent correlation.

Image compromise was defined as L3 CT slices where measurement of certain body composition parameters was not possible. This was assessed on a case‐by‐case and parameter‐by‐parameter basis, for example, in cases of ruptured AAA, where the extent of haematoma precluded accurate measurement of skeletal muscle area, yet it remained feasible to measure subcutaneous fat area. Where there was dubiety around the accuracy of body composition analysis, images were selectively excluded based on the compromised parameter.

ROC analysis was performed to assess the predictive value of each body composition parameter on both overall and 2‐year survival, which was chosen as a clinically relevant endpoint. To determine optimal thresholds for dichotomization of body composition parameters into ‘high’ and ‘low’, the ‘surv_cutpoint’ function of the ‘survminer’ R package was used, using the maximally selected rank statistic technique. Sex‐specific thresholds were derived to account for the established variation in body composition. For each body composition parameter, ROC curves were then visually inspected and thresholds derived by maximising sensitivity + specificity. These thresholds were compared to those generated by ‘surv_cutpoint’ and were each within a 5% difference, therefore the ‘surv_cutpoint’ values were used for subgroup analysis.

Differences between continuous variables were assessed using the Kruskal‐Wallis and Mann–Whitney Tests, and differences between categorical variables using the Chi‐Squared Test. Time‐to‐event analyses were calculated using the Kaplan–Meier method, with differences between cohorts assessed using the log‐rank t‐test. Where time to event survival data did not reach a median survival, the mean (95% CI) values are reported. *p* values < 0.05 were considered statistically significant. Linear regression was used to assess the relationship between continuous covariates. The effect of body composition on one and 5‐year mortality was assessed using binary logistic regression. The relationship between covariates and survival was assessed using a Cox Proportional Hazards Model; covariates were initially interrogated in univariate analysis and those with univariate *P* < 0.05 were included in a multivariate backwards conditional model with the threshold for removal set at *P* < 0.10. Fat and muscle body composition parameters were modelled separately with significant baseline clinical characteristics. Analyses were performed using R version 4.2.0 and IBM SPSS 28.0.

## Results

Six hundred seventy‐four patients (541 EVAR and 133 F/B‐EVAR) were eligible for inclusion into the study; 58 (8.6%) patients were female, and the mean (SD) age was 74.4 (6.8) years. Median (IQR) time from CT to surgery was 80 (103) days. All patients completed a minimum of 18 months follow‐up. When compromised images were excluded on a parameter‐by‐parameter basis, there were 605 cases (SFI), 641 cases (VFI), 666 cases (PMI), 647 cases (SMI), and 654 cases (SMD). Median (IQR) follow‐up was 60.0 (27.0) months, and there were 191 deaths during the follow‐up period (158 asymptomatic AAA, 7 symptomatic AAA, 26 ruptured AAA). Mean (95% CI) survival in the entire study population was 73.8 (71.1–76.6) months.

**Figure 1:**
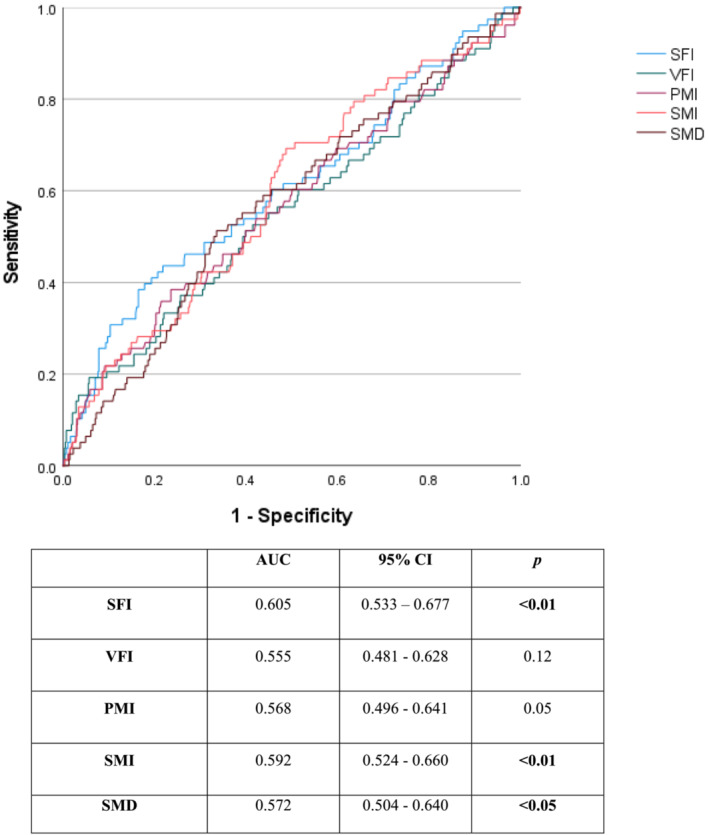
ROC curve and AUC values for the predictive value of body composition parameters on 2‐year survival in patients undergoing EVAR and F/B‐EVAR.

ROC curves describing the prognostic value of body composition parameters on 2‐year survival are shown in Figure 1. SFI (AUC 0.605), SMI (AUC 0.592), and SMD (AUC 0.572) provided prognostic value for 2‐year survival (*P* < 0.05); PMI and VFI did not provide significant prognostic value (*P* > 0.05).

Thresholds of body composition parameters to define the ‘low’ subgroups in male patients were: SFI 40.32 cm^2^/m^2^, VFI 58.61 cm^2^/m^2^, PMI 5.85 cm^2^/m^2^, SMI 49.69 cm^2^/m^2^, SMD 40.32 HU. Thresholds of body composition parameters to define the ‘low’ subgroups in female patients were: SFI 44.89 cm^2^/m^2^, VFI 59.36 cm^2^/m^2^, PMI 3.82 cm^2^/m^2^, SMI 37.73 cm^2^/m^2^, SMD 31.01 HU. When these thresholds were applied to the patient cohort, there were 81 patients (13.4%) with low SFI, 162 patients (25.3%) with low VFI, 167 patients (25.1%) with low PMI, 296 patients (45.7%) with low SMI, and 443 patients (67.7%) with low SMD.

**Figure 2:**
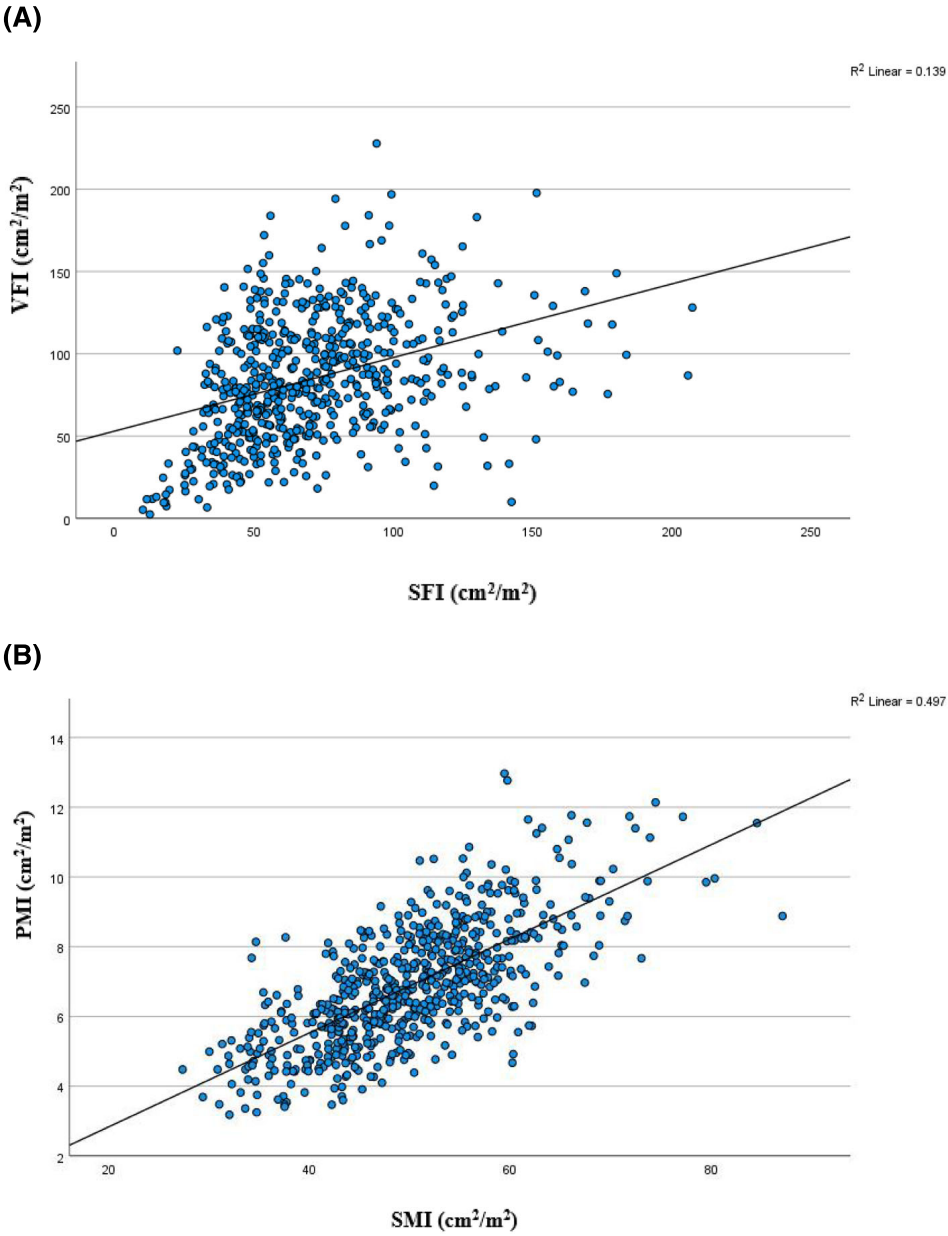
Scatter plots of (A) SFI (cm^2^/m^2^) versus VFI (cm^2^/m^2^) and (B) SMI (cm^2^/m^2^) versus PMI (cm^2^/m^2^) in patients undergoing EVAR and F/B‐EVAR with a linear interpolation line.

Figure 2A displays a scatter plot of SFI (cm^2^/m^2^) versus VFI (cm^2^/m^2^) with the interpolation line of best fit. Linear regression analysis produced an R^2^ value of 0.139. A scatter plot of SMI (cm^2^/m^2^) versus PMI (cm^2^/m^2^) with the interpolation line of best fit is displayed in Figure 2B. Linear regression analysis produced an R^2^ value of 0.497.

**Figure 3:**
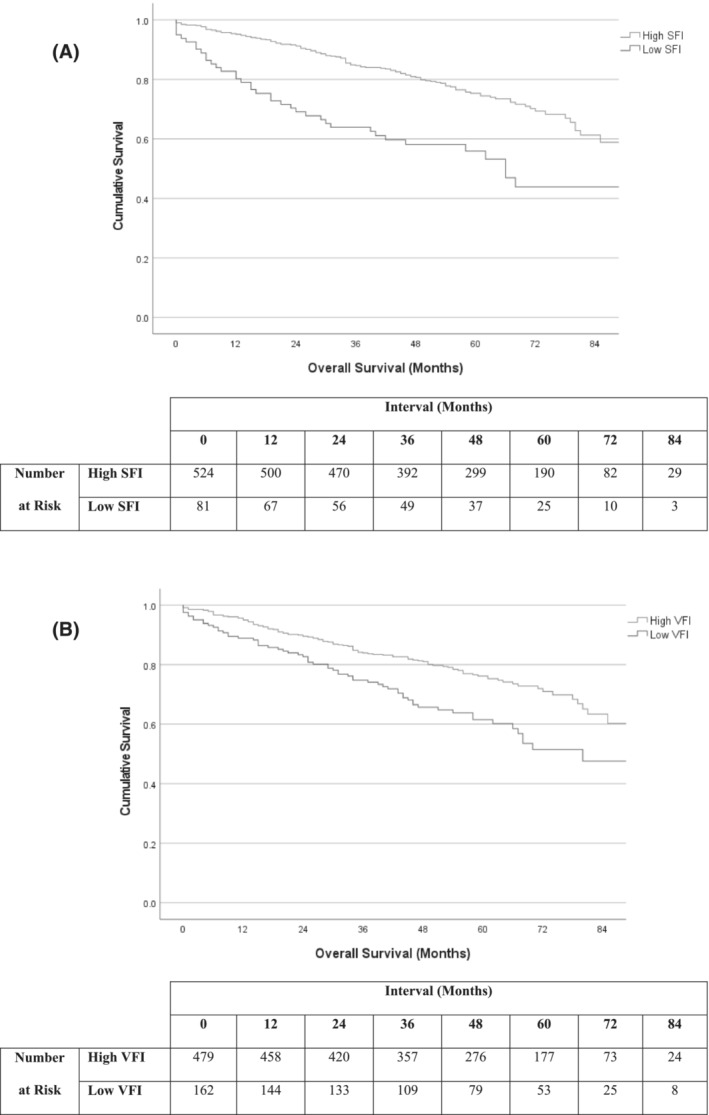
Overall survival in patients undergoing EVAR and F/B‐EVAR when sub‐grouped into (A) low and high SFI, *P* < 0.001 and (B) low and high VFI, *P* < 0.001.

**Figure 4:**
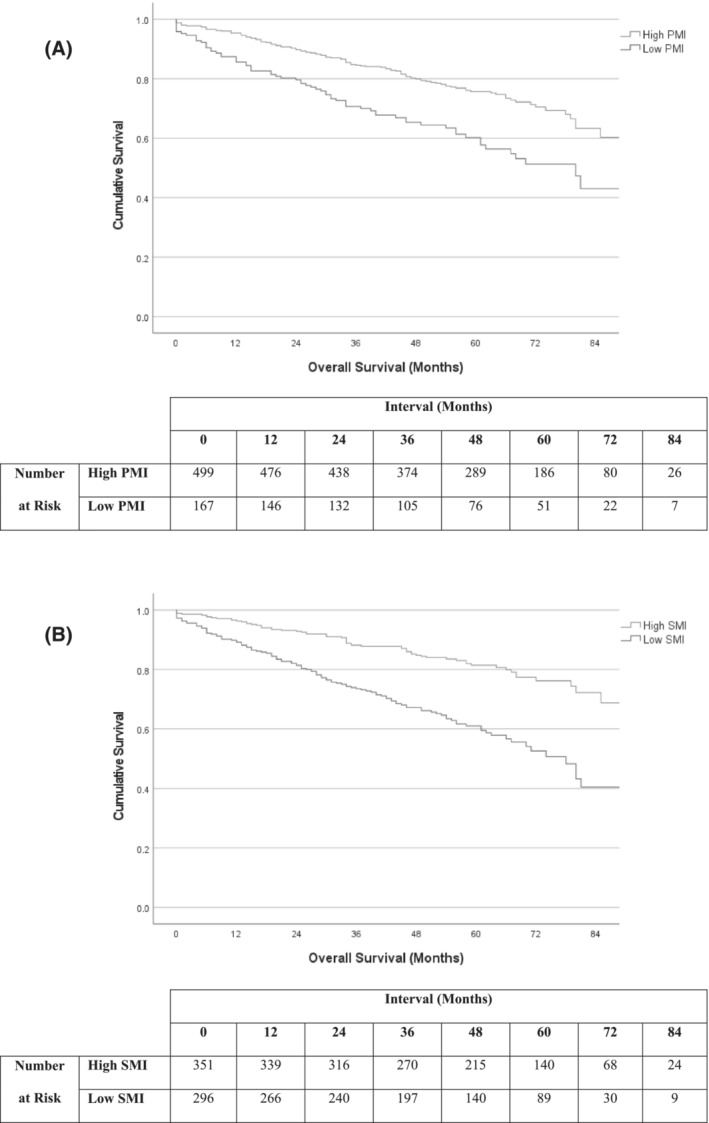
Overall survival in patients undergoing EVAR and F/B‐EVAR when sub‐grouped into (A) low and high PMI, *P* < 0.001 and (B) low and high SMI, *P* < 0.001.

Mean (95% CI) survival in the low SFI versus high SFI subgroups was 56.4 (48.2–64.7) versus 77.1 (74.2–80.1) months (*P* < 0.001, Figure 3A). Mean (95% CI) survival in the low VFI versus high VFI subgroups was 64.3 (58.8–69.8) versus 77.4 (74.3–80.5) months (*P* < 0.001, Figure 3B). Mean (95% CI) survival in the low PMI versus high PMI subgroups was 63.7 (57.7–69.7) versus 77.3 (74.3–80.4) months (*P* < 0.001, Figure 4A). Mean (95% CI) survival in the low SMI versus high SMI subgroups was 62.6 (58.5–66.7) versus 82.0 (78.7–85.3) months (*P* < 0.001, Figure 4B). Mean (95% CI) survival in the low SMD versus high SMD subgroups was 71.1 (67.7–74.6) versus 80.0 (75.7–84.3) months (*P* < 0.001).

Table 1 displays the univariate cox proportional hazards model describing the association between baseline clinical characteristics and body composition parameters, and overall survival. Ruptured AAA (HR 1.78, 95% CI 1.45–2.18, *P* < 0.001), increasing age (HR 1.98, 95% CI 1.52–2.58, *P* < 0.001), current tobacco smoking (HR 1.48, 95% CI 1.09–2.01, *P* < 0.05), low haemoglobin (HR 2.01, 95% CI 1.50–2.69, *P* < 0.001), high creatinine (HR 1.48, 95% CI 1.09–2.01, *P* < 0.05), low SFI (HR 2.35, 95% CI 1.63–3.37, *P* < 0.001), low VFI (HR 1.78, 95% CI 1.31–2.42, *P* < 0.001), low PMI (HR 1.94, 95% CI 1.44–2.61, *P* < 0.001), low SMI (HR 2.55, 95% CI 1.88–3.46, *P* < 0.001) and low SMD (HR 1.82, 95% CI 1.28–2.59, *P* < 0.001) were associated with inferior survival. High BMI (HR 0.77, 95% CI 0.64–0.93, *P* < 0.01) was associated with superior survival.

**Table 1:** The relationship between baseline clinical characteristics and pre‐operative CT‐derived body composition parameters and overall survival in patients undergoing EVAR and F/B‐EVAR (univariate model, *n* = 674)

Covariate	*N* (%)	HR	95% CI	*P*
Ruptured AAA	44 (6.5%)	1.78	1.45–2.18	<0.001
Age (<65/65–75/>75)	38 (5.6%)/338 (50.1%)/298 (44.2%)	1.98	1.52–2.58	<0.001
Female sex	58 (8.6%)	1.45	0.92–2.28	0.11
F/B‐EVAR	133 (19.7%)	0.83	0.57–1.21	0.33
Diabetes mellitus	112 (16.6%)	1.13	0.78–1.63	0.52
IHD	235 (35.0%)	1.17	0.87–1.57	0.30
Hypertension	452 (67.1%)	1.15	0.84–1.56	0.39
Prior CVA	85 (12.6%)	1.02	0.67–1.54	0.95
COPD	160 (23.7%)	1.20	0.87–1.65	0.28
Current tobacco smoker	162 (24.0%)	1.48	1.09–2.01	<0.05
Statin use	511 (76.0%)	0.74	0.54–1.02	0.67
BMI (<18.5/18.5–24.9/25.0–29.9/≥30.0 kg/m^2^)	6 (0.9%)/129 (19.3%)/288 (42.7%)/245 (36.4%)	0.77	(0.64–0.93)	<0.01
Low haemoglobin	167 (24.8%)	2.01	1.50–2.69	<0.001
High creatinine	174 (25.8%)	1.48	1.09–2.01	<0.05
Low SFI	81 (13.4%)	2.35	1.63–3.37	<0.001
Low VFI	162 (25.3%)	1.78	1.31–2.42	<0.001
Low PMI	167 (25.1%)	1.94	1.44–2.61	<0.001
Low SMI	296 (43.9%)	2.55	1.88–3.46	<0.001
Low SMD	443 (65.7%)	1.82	1.28–2.59	<0.001

Table 2 displays the multivariate backwards conditional cox proportional hazards model describing the association between baseline clinical characteristics and body composition parameters, and overall survival. Regarding body composition parameters, SMI and SFI were chosen for inclusion in the multivariate model based on their superior prognostic value on ROC and univariate analyses. Following stepwise removal of covariates; ruptured AAA (HR 1.46, 95% CI 1.10–1.93, *P* < 0.01), increasing age (HR 1.56, 95% CI 1.16–2.09, *P* < 0.01), current tobacco smoking (HR 1.42, 95% CI 1.00–2.00, *P* < 0.05), low haemoglobin (HR 1.42, 95% CI 1.01–2.00, *P* < 0.05), low SFI (HR 1.90, 95% CI 1.30–2.76, *P* < 0.001), and low SMI (HR 1.88, 95% CI 1.34–2.63, *P* < 0.001) were associated with inferior survival.

**Table 2:** The relationship between baseline clinical characteristics, pre‐operative CT‐derived SFI and SMI, and overall survival in patients undergoing EVAR and F/B‐EVAR (significant residual covariates from backwards conditional multivariate model, *n* = 674)

Covariate	HR	95% CI	*P*
Ruptured AAA	1.46	1.10–1.93	<0.01
Age (<65/65–75/>75)	1.56	1.16–2.09	<0.01
Current tobacco smoker	1.42	1.00–2.00	<0.05
Low haemoglobin	1.42	1.01–2.00	<0.05
Low SFI	1.90	1.30–2.76	<0.001
Low SMI	1.88	1.34–2.63	<0.001

Subgroup analysis was performed on the asymptomatic AAA (*n* = 604) cohort alone, in which there were 158 deaths during the follow‐up period. Table 3 displays the univariate cox proportional hazards model describing the association between baseline clinical characteristics and body composition parameters, and overall survival in patients with asymptomatic AAA. Increasing age (HR 2.07, 95% CI 1.54–2.77, *P* < 0.001), current tobacco smoking (HR 1.61, 95% CI 1.15–2.25, *P* < 0.01), low haemoglobin (HR 2.00, 95% CI 1.44–2.80, *P* < 0.001), high creatinine (HR 1.46, 95% CI 1.04–2.06, *P* < 0.05), low SFI (HR 1.98, 95% CI 1.32–2.97, *P* < 0.001), low VFI (HR 1.76, 95% CI 1.27–2.45, *P* < 0.001), low PMI (HR 1.73, 95% CI 1.24–2.40, *P* < 0.01), low SMI (HR 2.30, 95% CI 1.66–3.18, *P* < 0.001) and low SMD (HR 1.85, 95% CI 1.26–2.72, *P* < 0.01) were associated with inferior survival. High BMI (HR 0.72, 95% CI 0.59–0.88, *P* < 0.01) was associated with superior survival.

**Table 3:** The relationship between baseline clinical characteristics and pre‐operative CT‐derived body composition parameters and overall survival in patients undergoing EVAR and F/B‐EVAR for asymptomatic AAA (univariate model, *n* = 604)

Covariate	*N* (%)	HR	95% CI	*P*
Age (<65/65–75/>75)	31 (5.1%)/311 (51.5%)/262 (43.4%	2.07	1.54–2.77	<0.001
Female sex	48 (7.9%)	1.20	0.69–2.07	0.52
F/B‐EVAR	129 (21.4%)	0.85	0.57–1.27	0.44
Diabetes Mellitus	107 (17.7%)	1.13	0.77–1.68	0.53
IHD	217 (36.0%)	1.17	0.85–1.61	0.34
Hypertension	407 (67.4%)	0.94	0.67–1.30	0.69
Prior CVA	78 (13.0%)	0.95	0.60–1.50	0.81
COPD	145 (24.0%)	1.28	0.90–1.81	0.16
Current tobacco smoker	144 (24.0%)	1.61	1.15–2.25	<0.01
Statin use	473 (78.3%)	0.75	0.52–1.07	0.12
BMI (<18.5/18.5–24.9/25.0–29.9/≥30.0 kg/m^2^)	6 (1.0%)/116 (19.3%)/254 (42.2%)/226 (37.5%)	0.72	0.59–0.88	<0.01
Low haemoglobin	130 (21.7%)	2.00	1.44–2.80	<0.001
High creatinine	144 (24.0%)	1.46	1.04–2.06	<0.05
Low SFI	70 (12.5%)	1.98	1.32–2.97	<0.001
Low VFI	146 (24.5%)	1.76	1.27–2.45	<0.001
Low PMI	146 (24.3%)	1.73	1.24–2.40	<0.01
Low SMI	259 (44.3%)	2.30	1.66–3.18	<0.001
Low SMD	395 (66.8%)	1.85	1.26–2.72	<0.01

Table 4 displays the multivariate backwards conditional cox proportional hazards model describing the association between baseline clinical characteristics and body composition parameters (SFI and SMI), and overall survival in patients with asymptomatic AAA. Following stepwise removal of covariates; increasing age (HR 1.74, 95% CI 1.26–2.40, *P* < 0.001), current tobacco smoking (HR 1.60, 95% CI 1.11–2.31, *P* < 0.05), low haemoglobin (HR 1.59, 95% CI 1.11–2.28, *P* < 0.05), low SFI (HR 1.54, 95% CI 1.01–2.35, *P* < 0.05), and low SMI (HR 1.71, 95% CI 1.20–2.42, *P* < 0.01) were associated with inferior survival.

**Table 4:** The relationship between baseline clinical characteristics, pre‐operative CT‐derived SFI and SMI, and overall survival in patients undergoing EVAR and F/B‐EVAR for asymptomatic AAA (significant residual covariates from backwards conditional multivariate model, *n* = 604)

Covariate	HR	95% CI	*P*
Age (<65/65–75/>75)	1.74	1.26–2.40	<0.001
Current tobacco smoker	1.60	1.11–2.31	<0.05
Low haemoglobin	1.59	1.11–2.28	<0.05
Low SFI	1.54	1.01–2.35	<0.05
Low SMI	1.71	1.20–2.42	<0.01

The prognostic value of previously reported body composition thresholds was assessed by applying the thresholds for SMI and SMD proposed by Martin et al.^17^ to the AAA cohort in the present study. The prevalence of patients with low SMI and low SMD was 54.4% and 38.1% respectively. On univariate models, both SMI (HR 1.67, 95% CI 1.22–2.70, *P* < 0.01) and SMD (1.87, 95% CI 1.39–2.52, *P* < 0.001) were associated with inferior survival.

One‐year mortality in the low SMI versus high SMI subgroups was 10% versus 3% (*P* < 0.001). Low SMI was associated with increased odds of one‐year mortality (OR 3.19, 95% CI 1.60–6.34, *P* < 0.001). Five‐year mortality in the low SMI versus high SMI subgroups was 55% versus 28% (*P* < 0.001). Low SMI was associated with increased odds of five‐year mortality (OR 1.54, 95% CI 1.11–2.14, *P* < 0.01).

## Discussion

The present study demonstrates the prognostic value of pre‐operative CT‐derived body composition analysis in patients undergoing EVAR and F/B‐EVAR for AAA. Body composition analysis as a method of predicting mortality has been predominantly performed in patients with cancer, where there is a generally agreed consensus regarding measurement techniques and thresholds to define the pathological state. In contrast to this, body composition analysis in patients with AAA is in its relative infancy, with several studies reporting outcomes in patients undergoing EVAR.^20^, ^23^ While the majority of studies report inferior prognosis in patients with reduced muscle mass, this has not been reproduced by all authors.^23^ Most authors reporting outcomes in patients with AAA have reported patients with asymptomatic AAA only, with emergency cases and F/B‐EVAR underrepresented. Body composition analysis is more technically challenging in the setting of ruptured AAA, which is the likely justification for including asymptomatic cases only. The results in our unselected consecutive cohort represent a real‐world application of the use of body composition analysis and highlight the importance of further evaluation of this technique to develop a clinical tool for prognostication. Furthermore, the association observed by the present study was preserved in our subgroup analysis of asymptomatic AAA alone, strengthening the potential clinical application of body composition analysis.

SMD is a marker of myosteatosis and may reflect muscle function; there appears to be an association with clinically assessed muscle strength.^24^ The present study is the first to report SMD in patients undergoing EVAR, and the results are in keeping with findings from studies including patients with cancer.^17^

We also describe an association between SFI and VFI and prognosis in patients undergoing EVAR and F/B‐EVAR, with SFI appearing to offer superior prognostic value. Interestingly, our data describe only a weak correlation between SFI and VFI. To date, these parameters have not been reported with regards to prognosis in patients undergoing intervention for AAA. Charette et al. report that low VFI and SFI appear to confer inferior prognosis in patients with metastatic chemorefractory colorectal cancer.^16^ The mechanism by which fat indices confer prognosis is unclear and requires further validation in both external AAA and non‐AAA cohorts to accurately define this association. The so called ‘obesity paradox’ describes a controversial hypothesis that high BMI may be protective in a variety of conditions,^25^, ^26^ however may reflect reverse causality and inherent statistical bias. In patients with AAA, high BMI has not been identified as a clear risk factor for inferior prognosis,^27^ however in the present study higher BMI category predicted superior survival outcomes. Further interrogation of the complex interaction between BMI, body composition parameters, and postoperative prognosis is required.

There is general consensus that the superior prognostic marker of ‘radiological sarcopenia’ in patients with cancer is SMI,^28^ with very few authors reporting PMI in isolation. In contrast, there is no generally accepted superior muscle parameter for prognostication in patients with AAA, with psoas parameters predominantly reported to date. The results observed in the present study suggest that SMI offers superior prognostic value in both ROC and proportional hazards analyses. The linear association between SMI and PMI revealed only a fair association (R^2^ = 0.468), suggesting that despite the systemic nature of sarcopenia and cachexia, certain muscle groups may experience preferential muscle loss. This may relate to physical function and the use of psoas muscle as an aid to ambulation and maintaining posture. We suggest that future studies investigating body composition analysis in patients with AAA should use SMI, as it appears to be the superior parameter.

The use of validated thresholds of body composition parameters in patients with AAA has not yet been widely performed. Both data dependent thresholds (ROC, percentile) and absolute values of muscle area have been reported; absolute values are typically derived from cohorts of patients with cancer and lack validation in non‐cancer populations.^11^ There are established thresholds of body composition parameters derived from large cohorts of patients with cancer (Martin et al. and Xiao et al.^17^, ^18^). The thresholds derived for muscle analyses (SMI, SMD) in the present study were similar to those described by Martin et al., although the present study did not stratify thresholds based on BMI categories. Furthermore, when the thresholds proposed by Martin et al. were applied to the AAA cohort a high prevalence of sarcopenia, and prognostic value were observed, though this was inferior to the data‐derived thresholds. The prevalence of sarcopenia in patients with cancer appears to be similar irrespective of primary tumour origin and stage.^29^ The present study is the first to apply thresholds derived from patients with cancer to a non‐cancer population, with the novel observation that these thresholds may offer prognostic value irrespective of the nature of the underlying disease. It therefore appears that sarcopenia is prevalent across a range of chronic illnesses irrespective of the method used to define it, suggesting that there may be common host factors responsible for the development of sarcopenia, though there may be some disease‐specific variation. Increased recognition of host, and not just disease‐specific, factors contributing to the development of sarcopenia across a range of pathologies is required. Direct comparison of body composition between patients with cancer and patients with cardiovascular disease may allow for a greater understanding of the heterogeneity in sarcopenia between these patient groups.

The mechanism by which sarcopenia confers inferior prognosis remains poorly understood. A key component driving the progression of sarcopenia is thought to be chronic inflammation,^30^ which has also been identified as an aetiological factor in cardiovascular disease.^31^ Stent‐graft deployment in EVAR and F/B‐EVAR can induce an inflammatory response,^32^ which may contribute to increased long term mortality. Additionally, the association between body composition and functional physiological parameters is not widely reported, with only limited evidence available.^33^, ^34^ Reduced function and frailty are known to confer inferior prognosis in a range of conditions, and describing the relationship between body composition, function, and outcomes is an important area for further research.

The observations from the present study that patients with low SMI have a poorer survival are in keeping with findings in other non‐AAA populations such as cancer.^29^ CT‐derived body composition has been carried out extensively in patients with colorectal cancer with the consistent reporting of a low SMI being associated with poorer survival. Indeed, the HR associated with the relationship between low SMI and overall survival is similar to those reported by Dolan et al. in a colorectal cancer cohort using multiple thresholds of SMI.^35^ Given that there are common risk factors shared by AAA and colorectal cancer this raises the issue of whether low SMI is primarily secondary to the disease state or is primarily constitutional and related to lifestyle factors.^29^ Ideally, to tease out these competing factors, a comparison of CT‐BC parameters between age and sex matched healthy controls and patients with AAA would be carried out. However, ethical considerations of exposing apparently healthy individuals to ionising radiation precludes such a comparison. Alternatively, longitudinal studies may shed some light on the basis of a low SMI.^36^ Therefore, it would be of interest to carry out a longitudinal study of CT‐derived body composition in patients with AAA. Irrespective, the present results indicate that a low SMI has prognostic value in patients following EVAR and F/B‐EVAR independent of other potential disease related confounding factors.

The evidence for repairing AAA at the diameter threshold of 5.5 cm is widely accepted.^3^ However, the increased risk of mortality in a subset of patients may result in alteration of this treatment threshold in order to ameliorate the risk of post‐operative long‐term mortality. Recent data suggest a lower rupture rate than previously thought for untreated 5.5–6.0 cm AAA; rates as low as a 2.2% cumulative 3‐year rupture rate have been reported.^37^ This relatively modest risk of rupture may justify expectant management in high‐risk patients, highlighting a potential important clinical utility for body composition analysis.

## Limitations

The present study has a number of limitations inherent in retrospective cross‐sectional studies. In particular, the patient sample may be subject to bias since it was from a single geographical location and therefore may not be representative of all patients undergoing EVAR and F/B‐EVAR for AAA. However, the study cohort was relatively large, and the data collection was comprehensive.

## Conclusions

The results in the present study suggest that CT‐derived body composition analysis offers prognostic value in patients undergoing EVAR and F/B‐EVAR. We propose thresholds for subgrouping patients based on body composition parameters, requiring external validation. There may be an important role for pre‐operative body composition analysis in risk stratification of patients undergoing EVAR and F/B‐EVAR. Sarcopenia appears to be prevalent in both cancer and non‐cancer populations, and may be related to shared host‐specific factors.

## Conflict of interest

Nicholas Bradley, Amy Walter, Ross Dolan, Alasdair Wilson, Tamim Siddiqui, Campbell Roxburgh, Donald McMillan, and Graeme Guthrie declare that they have no conflict of interest. The authors declare that this manuscript complies with the Ethical guidelines for authorship and publishing in the *Journal of Cachexia, Sarcopenia and Muscle*.^38^
